# A Greek National Cross-Sectional Study on Myotonic Dystrophies

**DOI:** 10.3390/ijms232415507

**Published:** 2022-12-07

**Authors:** Georgios K. Papadimas, Constantinos Papadopoulos, Kyriaki Kekou, Chrisoula Kartanou, Athina Kladi, Evangelia Nitsa, Christalena Sofocleous, Evangelia Tsanou, Ioannis Sarmas, Stefania Kaninia, Elisabeth Chroni, Georgios Tsivgoulis, Vasilios Kimiskidis, Marianthi Arnaoutoglou, Leonidas Stefanis, Marios Panas, Georgios Koutsis, Georgia Karadima, Joanne Traeger-Synodinos

**Affiliations:** 1 1st Department of Neurology, Eginition Hospital, Medical School, National and Kapodistrian University of Athens, 11528 Athens, Greece; 2 Laboratory of Medical Genetics, Medical School, National and Kapodistrian University of Athens, “Ag. Sofia” Children’s Hospital, 11527 Athens, Greece; 3 Postgraduate Program in Biostatistics School of Medicine, National and Kapodistrian University of Athens, 11527 Athens, Greece; 4 Department of Neurology, University Hospital of Ioannina, University of Ioannina, 45500 Ioannina, Greece; 5 Department of Neurology, School of Medicine, University of Patras, 26504 Patras, Greece; 6 2nd Department of Neurology, “Attikon” University Hospital, National and Kapodistrian University of Athens, 12462 Athens, Greece; 7 1st Department of Neurology, AHEPA University Hospital, Aristotle University of Thessaloniki, 54636 Thessaloniki, Greece; 8 Department of Clinical Neurophysiology, AHEPA University Hospital, Aristotle University of Thessaloniki, 54636 Thessaloniki, Greece

**Keywords:** myotonic dystrophy, Steinert’s disease, proximal myotonic myopathy, cataract

## Abstract

Myotonic Dystrophies (DM, Dystrophia Myotonia) are autosomal dominant inherited myopathies with a high prevalence across different ethnic regions. Despite some differences, mainly due to the pattern of muscle involvement and the age of onset, both forms, DM1 and DM2, share many clinical and genetic similarities. In this study, we retrospectively analyzed the medical record files of 561 Greek patients, 434 with DM1 and 127 with DM2 diagnosed in two large academic centers between 1994–2020. The mean age at onset of symptoms was 26.2 ± 15.3 years in DM1 versus 44.4 ± 17.0 years in DM2 patients, while the delay of diagnosis was 10 and 7 years for DM1 and DM2 patients, respectively. Muscle weakness was the first symptom in both types, while myotonia was more frequent in DM1 patients. Multisystemic involvement was detected in the great majority of patients, with cataracts being one of the most common extramuscular manifestations, even in the early stages of disease expression. In conclusion, the present work, despite some limitations arising from the retrospective collection of data, is the first record of a large number of Greek patients with myotonic dystrophy and emphasizes the need for specialized neuromuscular centers that can provide genetic counseling and a multidisciplinary approach.

## 1. Introduction

Myotonic dystrophies (DM) are autosomal dominant, progressive, multisystemic disorders [1]. To date, two forms of myotonic dystrophy have been described. Myotonic dystrophy type 1 (DM1; Steinert’s disease, MIM#160900) is the most common muscular dystrophy in adulthood due to an unstable CTG expansion in the 3′ untranslated region of the myotonic dystrophy protein kinase (*DMPK*) gene (MIM#605377). Myotonic dystrophy type 2 (DM2; Proximal myotonic myopathy, MIM#602668) is caused by a CCTG repeat expansion in the first intron of the *CNBP* gene (MIM#115995). For both diseases, a toxic mRNA gain of function mechanism is proposed [1,2,3].

Both DM1 and DM2 are characterized by multisystemic manifestations involving the central nervous system, the heart, the gastrointestinal tract, the endocrine, and the respiratory system. DM1 presents with prominent distal muscle weakness and a spectrum of severity extending from severe congenital to minimally symptomatic late-onset forms. On the contrary, DM2 usually becomes evident in the sixth decade, and no cases with congenital presentation have been reported to date [1,4].

We herein present demographic, clinical, and genetic data of a Greek cohort of DM1 and DM2 cases diagnosed in two large academic centers over a period of 26 years between 1994–2020.

## 2. Results

**Figure 1:**
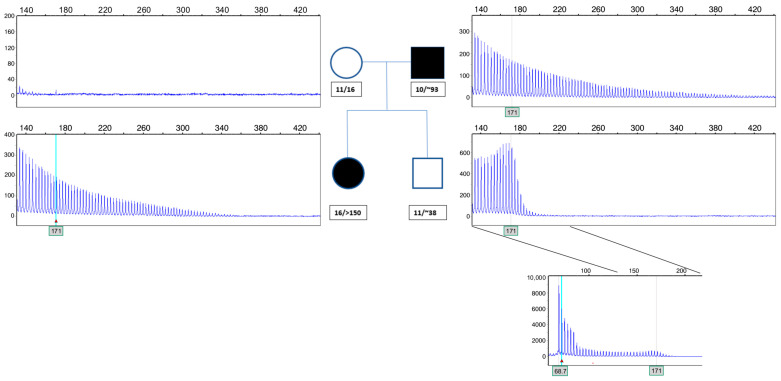
Segregation analysis revealed the contraction of an allele with ~100 repeats to 38 repeats when transmitted from a father to his asymptomatic son (family DM347).

In total, 561 patients, 434 (77.4%) with DM1 (209 males, 48.2% and 225 females, 51.8%) and 127 (22.6%) with DM2 (56 males, 44.1% and 71 females, 55.9%) were included in the present study (Table 1). Prenatal tests were recorded in 13 cases, of which three were found to have expansions. In one of the fetuses, a protomutation of 63 repeats originated from a maternal allele with 58 repeats. Expansions were also noticed in 17 families in the range of premutations (36–50 CTG repeats) or protomutations (51–80 CTG repeats) through transmission from unaffected parents. In another case, an allele of 100 repeats was contracted to 38 repeats (Figure 1). In total, from 30 parent–child pairs tested, 7 individuals were positive for premutations (4 families) and 25 for protomutations (13 families) (Table 2). In all cases of paternal transmission, expansions exceeded 150 repeats. Upon maternal transmission, only one expansion from 74 to >150 repeats was recorded, while the remaining three presented with a small expansion of up to 6 CTG repeats.

In the group of DM1 patients, the mean age of onset was 26.2 ± 15.3 years (Table 1) with non-significant differences between genders (23 (IQR; 15–37.5) male vs. 28.5 (IQR; 16–38) female, *p*-value = 0.3). Patients with maternal inheritance developed symptoms earlier than those with paternal inheritance (15.5 (IQR; 4–28.5) vs. 25 (IQR; 17–37), *p*-value < 0.001). The overall delay at diagnosis was 10 years (IQR; 4–19).

Among the DM1 patients, 134 (30.9%) recall muscular weakness as the first sign or symptom, 135 (31.1%) myotonia, 18 (4.1%) hypotonia (in congenital forms), and 7 (1.6%) had cataracts. For 110 patients, the first symptom is not recorded, whereas the remaining 30 patients reported other variable first symptoms (Table 3). Muscular weakness and myotonia developed over time in 315 (72.6%) and 275 (63.4%) patients, respectively. With respect to the cardiological complications, 83 (19.1%) patients had rhythm abnormalities and/or a pacemaker, and 21 (4.8%) had cardiomyopathy. Cataracts developed over time in 123 patients (28.3%), 47 patients (10.8%) had thyroid abnormalities, 35 (8.1%) suffered a respiratory syndrome, 26 (6.0%) had sleep apnea, 19 (4.4%) had diabetes mellitus, and 4 developed cancer (Table 4). Values of CPK and gamma-glutamyl transferase (GGT) were available in 85 and 32 patients, respectively, whereby elevated levels were detected in 30 patients (35.3%) with high CPK (223–980 IU/L) and 20 (62.5%) with high GGT (67–197 IU/L).

In the group of DM2 patients, the mean age of onset was 44.4 ± 17.0 years (Table 1) also with non-significant differences between genders (48 (IQR; 33–48) in males vs. 44 (IQR; 36–54) in females, *p*-value = 0.3). Data available from 55 patients showed paternal inheritance in 24 patients (18.9%) and maternal in 31 (24.4%). Patients with maternal inheritance developed symptoms later than those with paternal inheritance (45 (IQR; 38–54) vs. 30 (IQR; 20–47), *p*-value 0.02). The overall delay of diagnosis was 7 years (IQR; 3–18).

The first symptom, as reported by the patients, was muscle weakness for 79 (62.2%), myotonia for 12 subjects (9.4%), myalgia for 7 (5.5%), and cataracts for 2 (1.6%) (Table 3). However, 39 patients (30.7%) developed cataracts at a later stage of the disease (Table 3). Cardiological complications appeared over time in 10 patients (7.9%) with rhythm abnormalities and/or a pacemaker and 2 (1.6%) with cardiomyopathy. Concerning other health issues, 20 patients (15.7%) developed thyroid abnormalities, 19 (15.0%) diabetes mellitus, and 1 (0.8%) respiratory syndrome (Table 4). Interestingly, 39 patients had a mild to moderate serum CPK elevation (211–589 IU/L), and in 5 of them (3.9%), this was reported as the first sign of the disease randomly revealed in a routine check-up.

## 3. Discussion

**Figure 2:**
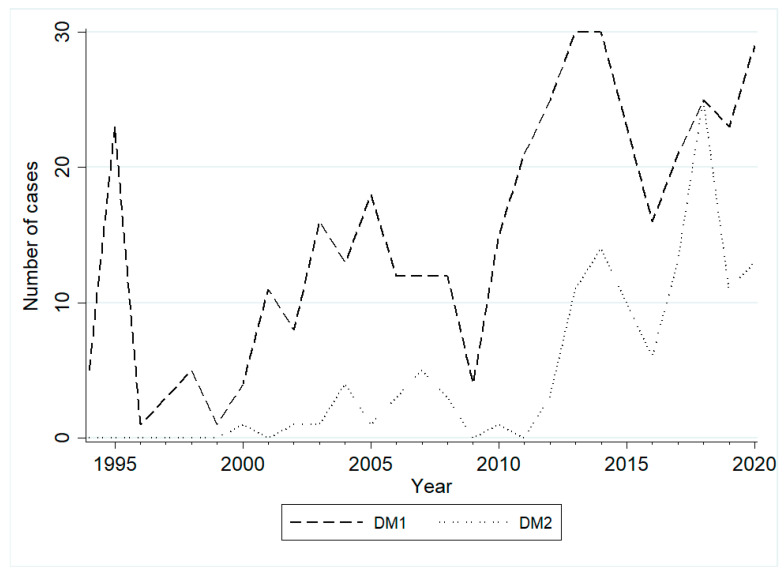
Number of cases diagnosed annually in both DM1 and DM2 patients.

This is the first large Greek retrospective cross-sectional study of patients with myotonic dystrophies. DM1 is considered the most common inherited myopathy in adults, with an estimated prevalence of 0.5 to 18.1 per 100,000 individuals worldwide [5]. The disease was initially described by Steinert in 1909, and the gene defect was discovered in 1992 [6]. Since it was only in the late 1990s that DM2 was recognized as a different form of myotonic dystrophy, concrete epidemiological data are still missing [7,8]. Nevertheless, early evidence suggests that the frequency of DM2 may be equal to DM1 or even higher in some areas, as shown in Northern European countries such as Finland and Germany [9,10]. As proof of the high incidence of DM2 in Greece as well, 137 (63.7%) of the 215 total new diagnoses since 2015 were DM1 and 78 (36.3%) DM2 (Figure 2). However, DM2 is probably underestimated because it often remains unrecognized due to mild and unspecific symptoms. Interestingly, DM1 patients with maternal inheritance developed symptoms earlier than those with paternal inheritance (15.5 years (IQR: 4–28.5) vs. 25 years (IQR: 17–37), *p* < 0.001), which is in accordance with previous studies reporting earlier onset and more severe symptoms when the disease is inherited from the mother [11]. In contrast, DM2 patients with maternal inheritance developed symptoms later than those with paternal inheritance, but this finding does not reach statistical significance (45 (IQR; 38–54) vs. 30 (IQR; 20–47), *p*-value = 0.2).

In accordance with previous studies, paternally transmitted protomutations (<80 repeats) in our cohort were noted to be far more unstable, leading to both expansions (12/13) and rare contractions (1/13), than those maternally inherited (1/4). The above finding is in accordance with previous studies and implies that males with less than 80 repeats have a higher risk of symptomatic offspring [12,13]. On the other hand, congenital forms of DM1 were recorded only through maternal inheritance of mutations (>150 repeats) from affected mothers. The maternal inheritance in most congenital DM1 cases is well described, but the underpinning mechanisms seem to be complex and not fully elucidated. The inability of sperms with large CTG expansions to fertilize oocytes and epigenetic factors regulating gene expression and methylation/demethylation have been implicated as possible etiologies [14]. In the context of genetic counseling and the risk for symptomatic offspring, these patterns of inheritance call for special management, including the possible need for prenatal or preimplantation genetic diagnosis and proper interpretation.

The age range of disease onset differs between DM1 and DM2. The adult form of DM1 develops in the 3rd and 4th decades of life, while there are also juvenile, childhood, and congenital forms [2]. On the contrary, DM2 presents mainly in adulthood and lacks a congenital form [15]. In accordance with the above, most DM2 patients in our study had their first symptom in adulthood, although a measurable proportion developed some signs of the disease even before the age of 20. On the other end of the spectrum, a few patients presented their initial symptoms at an advanced age, emphasizing that there are also very late-onset forms that might occasionally be indistinguishable from the effects of normal aging. The mean diagnostic delay was 10 and 7 years for DM1 and DM2, respectively, which could be mainly attributed to the fact that in the past, there were few neuromuscular centers across the country, and most physicians were not familiar with the clinical signs and symptoms of these disorders. Furthermore, especially for DM2, molecular diagnostics was not available until about 2010.

Although muscle weakness is a prominent feature of both entities, a cardinal clinical distinction between the two types of myotonic dystrophy is the prominent distal versus proximal distribution of muscle weakness in DM1 and DM2, respectively [16]. Moreover, myotonia was infrequently the first symptom of the disease in the DM2 patients of the present cohort, in contrast to DM1 patients, where myotonia and muscle weakness were equally frequent as primary symptoms (Table 3). Myotonia, despite being a key feature of both conditions, is more frequent in DM1 with fingers, tongue, and jaw being mainly affected, while in DM2, it can be mild without interfering with daily activities [16]. This is in line with the findings from our cohort, where clinical myotonia was observed in a substantially higher proportion of DM1 patients. In fact, myotonic discharges may be subtle or even absent in the EMG of DM2 patients [17].

Myalgia was also a very common complaint in DM2 patients of the present study, in contrast to DM1 patients who did not report such a symptom. Muscle pain may be observed in various neuromuscular disorders but seems to be an early and frequent symptom in DM2. In a study of patients with myotonic dystrophy in Finland, muscle pain, although more common in DM2, was also quite common in DM1 [18]. Similarly, a retrospective German survey reported a substantially higher frequency of muscle pain in DM2 patients either at the onset or at a later stage of the disease [19]. The nature of the pain may be highly variable, from a dull ache or stiffness to a pain resembling fibromyalgia, and can be aggravated by exercise and low temperature [20,21].

From the extramuscular symptoms, an early onset posterior subcapsular cataract was one of the most common and sometimes even the primary manifestation of the disease in both types [22], which was also confirmed in the present study. This is in agreement with two previous small Greek cohorts: one with DM2 patients, which showed that 32.1% of them were diagnosed with an early onset cataract at a median age of 43 years, and for 25%, it was the first symptom of the disease [23] and another with DM1 patients, where Christmas tree cataracts were reported in 56% of individuals and was the first sign for almost half of them [24]. However, it should be emphasized that the percentage of cataracts in both groups of the present study is certainly far lower than expected due to the missing data.

The incidence of heart abnormalities is high in myotonic dystrophies, and cardiac arrhythmias may occasionally be the presenting manifestation of the disease, even earlier than the onset of muscular symptoms [25]. Cardiac involvement, mainly in the form of EKG changes and rhythm disturbances, was more common in DM1 patients of the present study. Respiratory insufficiency, due to peripheral and central pathway involvement, remains the leading cause of death in adult-onset DM1 patients [26,27]. Moreover, DM1 is well known to be associated with sleep disturbances, and there are also recent studies supporting a high incidence of sleep disorders even in DM2 [28,29,30]. A significantly higher percentage of DM1 patients in the present cohort had some degree of restrictive respiratory insufficiency and sleep apnea syndrome. However, since fewer DM2 patients were submitted to sleep studies, some of them may have simply missed the diagnosis of a sleep disorder. A variety of gastrointestinal symptoms are also frequently reported as part of the multisystemic myotonic dystrophies [2,19]. In a recent cohort of DM1 patients, the frequency of gastrointestinal manifestations and mainly constipation was high with a female preponderance, while serum GGT levels were more elevated in males [31]. Digestive symptoms, although not systematically recorded in the present study, seem to be quite common in DM1 patients, whereas they are neither frequent nor severe in DM2 patients. Notably, dysphagia was reported in a significant number of DM1 patients, increasing morbidity and impairing quality of life. Serum GGT levels were also increased in a high proportion of DM1 patients regardless of gender. Endocrine abnormalities, such as thyroid dysfunction, diabetes, or hypogonadism, are well described, especially in patients with DM1, and may occasionally be the presenting manifestations of the disease [2,20,32]. Thyroid dysfunction and mainly hypothyroidism was the most common endocrine disturbance in the present cohort in both types of myotonic dystrophy, followed by diabetes. Of note, thyroid disease has a prevalence of 9% in Greece according to a national health examination survey [33] versus 10.8% of DM1 and 15.7% of DM2 patients in the present study even despite the large missing data proportion.

Many previous studies imply an elevated cancer risk in patients with DM1, while similar data for DM2 are still lacking. The most common neoplasms associated with the classic DM1 are skin cancers and especially basal cell carcinoma, while other cancer types are found at a higher incidence in patients with DM1 [34,35]. Finally, evidence of cognitive deficit is present in a great proportion of patients with myotonic dystrophy and especially DM1, with a pronounced nonverbal episodic memory impairment, and the above observations are well associated with the results of brain imaging studies revealing hippocampal volume reduction and extensive white matter changes [36]. Unfortunately, data from a thorough neuropsychological assessment are not available for this cohort of patients to allow comparisons with existing literature.

The current study has many strengths and drawbacks. The main advantage is the large size and representativeness of the sample. On the other hand, the major limitation is the great number of missing data due to the retrospective nature of the study and the lack of homogeneity in data collection, partly because the patients are followed up in different centers. The latter also underlines the urgent need for a National Registry for Myotonic Dystrophy, especially in the era of new evolving treatments. This Registry should cover the whole country and be able to serve as a data source for the evaluation of specific outcome measures, but also for research, clinical and policy purposes.

In conclusion, the results of this study on Greek patients with DM1 and DM2 are broadly in agreement with the findings of previous large studies in other populations. Muscle weakness and myotonia are cardinal clinical features of both forms, although the symptoms of DM2 can sometimes be so mild that in the absence of high clinical suspicion, the diagnosis may be easily missed. Moreover, since both types of myotonic dystrophy are multisystemic disorders, there is a need for a multidisciplinary approach from healthcare providers with a deep knowledge of the diseases. Genetic counseling should be offered to all patients and their families, and despite the lack of treatment so far, it must be stressed that adherence to the standards of care has a significant impact on the quality of life and substantially decreases morbidity and mortality.

## 4. Materials and Methods

This retrospective analysis is restricted to data mined for statistical purposes and/or phenotype–genotype correlations from a total of genetically confirmed 561 patients with myotonic dystrophy. The study was approved by the Scientific and Ethics Committees of both centers, namely the Eginition University Hospital (No 515/05-10-2015) and “Aghia Sophia” Children’s Hospital (No 26935/19-12-2019). Since all data were de-identified and stored in an electronic file with numeric codes, no additional consent was required [37]. Molecular findings were provided by the 1st Department of Neurology (Neurogenetics Unit) and the Laboratory of Medical Genetics, both departments of the National and Kapodistrian University of Athens (NKUA). Informed consent for genetic analysis was provided either directly by all indiv37iduals tested (or their legal guardians) or through the referring clinicians.

Genotyping of CTG and CCTG repeat expansions in *DMPK* and *CNBP* genes, respectively, was based on the detection of repeats within the different size ranges rather than on the calculation of the exact number of repeats. A 2-step procedure was followed to include a first fluorescent polymerase chain reaction (haplotype PCR) and a second repeat-primed PCR (RP-PCR) [38]. Haplotyping allows the exclusion of expansions in the presence of two normal alleles as well as the detection of *DMPK* premutations or protomutations. *DMPK* alleles, with 36 up to 50 repeats, are termed as premutations, and from 51 up to 80 as protomutations. Samples with one allele were further analyzed with RP-PCR to evaluate the presence of larger expansions. In the case of *CNBP*, the detection of products over 550 bp reflects the presence of at least the lowest causative expansion of 75 repeats and is considered sufficient to set the diagnosis [39,40]. The first thirty samples of DM1 were genotyped for the CTG expansion using Southern blot analysis according to the procedure of Shelbourne et al. [41].

## 5. Statistical Analysis

Statistical analysis was performed using the statistical software STATA version 15.0 (Stata Corporation, College Station, TX, USA). Descriptive statistics were calculated for all variables. Number (Percentage) and Mean ± Standard Deviation (SD) or Median (IQR: 25th–75th) for categorical and continuous variables with normal or non-normal distribution, respectively. Mann–Whitney U test was used to compare the age of onset with the gender, the parental origin of expansion, and the year of diagnosis. A two-sided *p*-value < 0.05 was assigned as the threshold for statistically significant findings.

## Figures and Tables

**Table 1:** Main characteristics of subjects.

	Myotonic Dystrophy Type 1	Myotonic Dystrophy Type 2
Total (N (%))	434 (100.0)	127 (100.0)
Gender (N (%))		
Male	209 (48.2)	56 (44.1)
Female	225 (51.8)	71 (55.9)
Age of onset in years (mean ± SD)	27.1 ± 14.7	44.4 ± 17.0
Diagnostic delay in years (Median (IQR; 25–75))	8 (2–17.5)	6 (2–17)

**Table 2:** Premutations, protomutations, and full-sized mutations detected in subjects referred for DM1.

Parental Origin *	DM1 Premutations 36–50 CTG Repeats	DM1 Protοmutations 51–80 CTG Repeats	Full-Sized DM1 Mutations >80 CTG Repeats
Paternal	1 (contraction)	3	118
Maternal	2	9 + 1 fetus	101
Unknown	4	13	183
Total	7	25	402

* Parental origin was evaluated through molecular analysis or based on the family trees.

**Table 3:** Presenting symptoms in Myotonic Dystrophy Type 1 and Type 2.

Symptom	Myotonic Dystrophy Type 1 N (%)	Myotonic Dystrophy Type 2 N (%)
Muscle Weakness	134 (30.9)	79 (62.2)
Myotonia	135 (31.1)	12 (9.4)
Hypotonia	18 (4.2)	0 (0.0)
Cataract	7 (1.6)	2 (1.6)
Dysarthria	6 (1.4)	0 (0.0)
Cardiological complications	4 (0.9)	0 (0.0)
Cognitive difficulties	4 (0.9)	0 (0.0)
Dysphagia	4(0.9)	0 (0.0)
Nasal speech	3 (0.7)	0 (0.0)
Fatigue	2 (0.5)	0 (0.0)
Ptosis	2 (0.5)	0 (0.0)
Pes valgus	1 (0.2)	0 (0.0)
Respiratory complications	1 (0.2)	0 (0.0)
Hypogonadism	1 (0.2)	0 (0.0)
Rhabdomyolysis	1 (0.2)	0 (0.0)
Cognitive impairment	1 (0.2)	0 (0.0)
Myalgia	0 (0.0)	7 (5.5)
Increased CPK	0 (0.0)	5 (3.9)
Somnolence	0 (0.0)	2 (1.6)
Stiffness	0 (0.0)	2 (1.6)
Cramp	0 (0.0)	2 (1.6)
Fatigue	0 (0.0)	1 (0.8)
Unknown	110 (25.4)	15 (11.8)
Total	434 (100.0)	127 (100.0)

**Table 4:** Comorbidities in Myotonic Dystrophy Type 1 and Type 2.

	Myotonic Dystrophy Type 1	Myotonic Dystrophy Type 2
Comorbidity (N (%))	434 (100.0)	127 (100.0)
Muscle weakness Yes No Unknown	315 (72.6) 26 (6.0) 93 (21.4)	96 (75.6) 14 (11.0) 17 (13.4)
Myotonia Yes No Unknown	275 (63.4) 25 (5.8) 134 (30.9)	75 (59.1) 10 (7.9) 42 (33.1)
Fatigue Yes No Unknown	68 (15.7) 63 (14.5) 303 (69.8)	35 (27.6) 16 (12.6) 76 (59.8)
Cataract Yes No Unknown	123 (28.3) 124 (28.6) 187 (43.1)	39 (30.7) 49 (38.6) 39 (30.7)
Rhythm abnormalities/and or pacemaker Yes No Unknown	83 (19.1) 129 (29.7) 222 (51.2)	10 (7.9) 73 (57.5) 44 (34.6)
Cardiomyopathy Yes No Unknown	21 (4.8) 128 (29.5) 285 (65.7)	2 (1.6) 81 (63.8) 44 (34.6)
Respiratory inv Yes No Unknown	35 (8.1) 80 (18.4) 319 (73.5)	1 (0.8) 27 (21.3) 99 (77.9)
Daytime sleepiness Yes No Unknown	25 (5.8) 63 (14.5) 346 (79.7)	7 (5.5) 0 (0.0) 120 (94.5)
Sleep apnea Yes No Unknown	26 (6.0) 48 (11.1) 360 (82.9)	0 (0.0) 12 (9.4) 115 (90.6)
Ventilation Yes No Unknown	15 (3.5) 72 (16.6) 332 (76.5)	0 (0.0) 0 (0.0) 127 (100.0)
Thyroid dysfunction Yes No Unknown	47 (10.8) 157 (36.2) 230 (53.0)	20 (15.7) 57 (44.9) 50 (39.4)
Glucose intolerance Yes No Unknown	19 (4.4) 181 (41.7) 234 (53.9)	19 (15.0) 48 (37.8) 60 (47.2)
Dysphagia Yes No Unknown	37 (8.5) 65 (15.0) 332 (76.5)	0 (0.0) 11 (8.7) 116 (91.3)
Cancer Yes No Unknown	4 (0.9) 216 (49.8) 214 (49.3)	12 (9.4) 5 (3.9) 110 (86.7)

## Data Availability

The datasets included and/or analyzed during the current study are available from the Neurogenetics Unit (1st Department of Neurology) and the Laboratory of Medical Genetics, both departments of the National and Kapodistrian University of Athens (NKUA), on reasonable request.
